# Social Protection Interventions for TB-Affected Households: A Scoping Review

**DOI:** 10.4269/ajtmh.22-0470

**Published:** 2023-02-20

**Authors:** Heather Todd, Mollie Hudson, Natalia Grolmusova, Joseph Kazibwe, Joseph Pearman, Kristina Skender, Phuong B. Tran, Delia Boccia, Priya B. Shete, Tom Wingfield

**Affiliations:** ^1^ Departments of Clinical Sciences and International Public Health, Liverpool School of Tropical Medicine, Liverpool, United Kingdom;; ^2^ School of Medicine, University of Liverpool, Liverpool, United Kingdom;; ^3^ School of Nursing, University of California San Francisco, San Francisco, California;; ^4^ Department of Global Public Health, World Health Organization Collaborating Centre on Tuberculosis and Social Medicine, Karolinska Institutet, Stockholm, Sweden;; ^5^ London School of Hygiene and Tropical Medicine, Liverpool, United Kingdom;; ^6^ Department of Clinical Sciences, Lund University, Malmö, Sweden;; ^7^ Department of Family Medicine and Population Health, University of Antwerp, Antwerp, Belgium;; ^8^ Center for Tuberculosis University of California, San Francisco, San Francisco, California;; ^9^ Division of Pulmonary and Critical Care Medicine, San Francisco General Hospital, San Francisco, California;; ^10^ Tropical and Infectious Diseases Unit, Liverpool University Hospitals NHS Foundation Trust, Liverpool, United Kingdom

## Abstract

Tuberculosis (TB) and poverty are inextricably linked. Catastrophic costs of TB illness drive TB-affected households into worsening impoverishment and hamper treatment success. The WHO’s End TB Strategy recommends social protection for TB-affected households to mitigate financial shock and improve TB outcomes. This scoping review maps the landscape of social protection interventions for people with TB and their households in low- and middle-income countries with high TB burden. A systematic search of Medline, Embase, PubMed, and Web of Science for relevant articles was performed, supplemented with a gray literature search of key databases. Articles were included if they described social protection available to people with TB and TB-affected households in a low- or middle-income country. Data were synthesized in tabular form, and descriptive narrative outlined the successes and challenges of the social protection interventions identified. The search identified 33,360 articles. After abstract screening, 74 articles underwent full text screening, and 49 were included in the final analysis. Forty-three types of social protection were identified, of which 24 were TB specific (i.e., only people with TB were eligible). Varying definitions were used to describe similar social protection interventions, which limited cross-study comparison. Intervention successes included acceptability and increased financial autonomy among recipients. Challenges included delays in intervention delivery and unexpected additional bank transfer fees. A wide range of acceptable social protection interventions are available, with cash transfer schemes predominating. Use of standardized definitions of social protection interventions would facilitate consolidation of evidence and enhance design and implementation in future.

## INTRODUCTION

Tuberculosis remains a global public health priority. It is predicted that there will be a 20% increase in TB deaths over the coming 5 years due to the impact of COVID-19.^1^ TB not only has devastating health implications but also severe socioeconomic impacts on those affected and their households. This socioeconomic burden falls mostly on poorer and vulnerable people and communities in low- and middle-income countries and perpetuates a cycle of poverty.^2^ An analysis by Silva et al. suggested that delays in achieving the World Health Organization’s 2015 End TB Strategy goals to reduce TB prevalence and mortality would have profound economic and health consequences, which would disproportionately affect sub-Saharan African countries.^3^ The major drivers of TB are undernutrition, poverty, diabetes, tobacco smoking, and household air pollution,^4^ which contribute to nearly half of the global TB burden. Such determinants need to be addressed urgently, including through social protection.^5^

When identified and treated early, TB is curable. However, there remain numerous challenges to completing treatment and achieving prolonged cure. The standard treatment regimen for drug-sensitive TB (DS-TB) lasts for 6 months. Drug-resistant TB (DR-TB) regimens are arduous, toxic, can involve injectable agents, can extend up to 24 months.^6^^,^^7^ The socioeconomic impacts of DS-TB and especially DR-TB are severe. Although all age groups are at risk, TB tends to affect adults in their most economically productive years. The economic devastation associated with loss of income and productivity, unemployment, and out-of-pocket medical costs (e.g., consultations, tests, pills) and nonmedical costs (e.g., food, travel) can make even “free” TB care expensive.^8^ Catastrophic costs (defined as total TB-related costs > 20% of a TB-affected household’s annual pre-TB income) can reduce the capacity of a household to cope with financial shocks and hamper access to and completion of TB treatment.^8^ According to the 2021 Global TB report, 45% and 87% of DS-TB and DR-TB-affected households incur catastrophic costs.^9^

The TB poverty cycle can be interrupted using social protection measures that alleviate poverty, reduce food insecurity and mitigate catastrophic costs of TB-affected households.^10^^,^^11^ Previous systematic reviews have examined the impact of social protection on the social determinants of TB.^10^^–^^13^ However, breadth and depth of exploration has been constrained by a limited evidence base and variable definitions or scope of what constitutes social protection. Moreover, since the introduction of the End TB strategy in 2015, which emphasized the importance of addressing the social determinants and consequences of TB and included a global Catastrophic Costs indicator, there has been a notable expansion of new evidence in the field.

This scoping review (ScR) will provide a much-needed assessment of the social protection interventions and programs available for people with TB and their households and evaluate the challenges and successes of their implementation including intervention design, recipients, and logistics of access, delivery, and receipt.

The aims of this ScR were to 1) establish what social protection interventions are available to people with TB and their households in low- and middle-income and/or TB high-burden countries, 2) describe the successes and challenges of implementation and delivery of available social protection interventions, and 3) inform the design of a systematic review and meta-analysis of the impact of social protection on TB and socioeconomic outcomes.

## METHODS

To facilitate future similar reviews or related comparative reviews, this ScR used the recognized World Bank definition of social protection being “systems which seek to improve inequalities and reduce intergenerational poverty by seeking to help individuals and families, especially the poor and vulnerable, cope with crises and shocks, find jobs, improve productivity, invest in the health and education of their children and protect the aging population.”^14^ This umbrella term refers to interventions that include but are not limited to cash transfers, vouchers, food baskets, and nutritional supplementation.

The ScR was guided by the Arksey and O’Malley guidance and PRISMA ScR (PRISMA-ScR) extension checklist, which suggest inclusive search strategies of both the published and gray literature.^15^^–^^17^ A literature search was carried out in the research databases PubMed, Embase, Medline and Web of Science on March 10, 2021 (see supplemental materials for full search strategies). The eligibility criteria were defined using the PICOT approach outlined in Table 1. With regard to outcomes, the scoping review focused primarily on process outcome measures including successes and challenges of social protection interventions. As per a key aim of the ScR, impact outcome measures such as TB treatment outcome and catastrophic costs were also included as part of the eligibility criteria to identify studies with quantitative outcome data suitable for inclusion in a related systematic review and meta-analysis of the impact of social protection on TB and broader socioeconomic outcomes.

**Table 1:** PICOT inclusion criteria

Population	People with tuberculosis (TB) or living in TB-affected households (e.g., one or more household members with TB disease) were included. Countries classified by the World Bank as TB high-burden countries and/or low- and middle-income countries at the time the study was conducted were included. Countries classified as high income and/or non–high burden at the time of study were excluded.
Intervention	Standard TB care and access to at least one social protection intervention or program. In this context, access is defined as the receipt of services, not just the existence of a social protection intervention or program in a given area.
Control	People with TB receiving standard TB care who did not have access to any social protection intervention or program.
Outcomes	Challenges and successes of the implementation of social protection interventions, which included but were not limited to reported uptake, fidelity, feasibility, and acceptability. TB treatment outcomes were in line with updated WHO recommendations^18^ and included TB treatment success (cure or treatment completion) or adverse TB treatment outcomes (loss to follow-up, death, treatment failure, and relapse) and catastrophic costs (total costs of entire TB illness > 20% of the same household’s annual pre-TB income). Socioeconomic outcomes included catastrophic costs and dissaving (coping strategies to absorb financial shock including but not limited to reduced household food consumption^19^ using savings, taking out formal or informal loans, selling assets, taking children out of school, seeking additional employment or income, and selling sex). Other socioeconomic outcomes included multidimensional poverty indices, household crowding, and food insecurity.
Time	All studies published from 2012 to present were considered. The time frame for study eligibility is based on the “World Bank’s Social Protection and Labour Strategy 2012–2022,” in which the World Bank focused their initiatives on reducing socioeconomic risk and strengthening social protection programs. Expanding the date range beyond this would have resulted in a yield of studies too great to manage.

**Table 2:** Characteristics of included studies

Author (year)	Study design	Outcomes	Country	Setting	TB HBC	Income classification
Ciobanu et al.^21^	Cohort	Treatment success; number of people with TB receiving incentives; types of incentives among those who received them	Moldova	Mixed	No	LMIC
Ukwaja et al.^22^	Cohort	Treatment success; determinants of successful outcomes	Nigeria	Rural	Yes	LMIC
Oliosi et al.^23^	Cohort	Treatment outcomes	Brazil	Urban	Yes	UMIC
Torrens et al.^24^	Cohort	Treatment success	Brazil	Mixed	Yes	UMIC
Rohit et al.^25^	Cohort	Treatment outcomes	India	Mixed	Yes	LMIC
Priedeman Skiles et al.^26^	Cohort	Loss to follow-up; program impact on treatment default*	Ukraine	Mixed	Yes	LMIC
Klein et al.^27^	Cohort	Treatment success	Argentina	Urban	Yes	UMIC
Malacarne et al.^28^	Case–control	Treatment success	Brazil	Peri-urban	Yes	UMIC
Bhavesh et al.^29^	Cohort	Utilization of social protection program; treatment success	India	Urban	Yes	LMIC
Mansour et al.^30^	Cohort	Lost to follow-up (defined as unable to be located, never started treatment after diagnosis confirmed or treatment interrupted after > 2 months)	Kenya	Mixed	Yes	LMIC
Bhatt et al.^31^	Cohort	Treatment success	India	Urban	Yes	LMIC
Samuel et al.^32^	Cohort	Treatment success	India	Mixed	Yes	LMIC
Durovni et al.^33^	Cohort	Treatment outcomes	Brazil	Urban	Yes	UMIC
Rudgard et al.^34^	Cross-section survey	Financial hardship†	Brazil	Urban	Yes	UMIC
Chirico et al.^35^	Case control	Clinical and epidemiological differences between people with TB included versus not included in the social protection regimen; treatment success	Argentina	Urban	Yes	UMIC
Zhao et al.^36^	Observational	Financial burden of transportation; recipient’s perceptions of social protection intervention	China	Rural	Yes	UMIC
Soares et al.^37^	Observational	Treatment success	Brazil	Urban	Yes	LMIC
Kaliakbarova et al.^38^	Observational	Treatment success; recipient satisfaction with social protection program	Kazakhstan	Urban	Yes	UMIC
Rogers et al.^39^	Cohort	Treatment success	Liberia	–	No	LIC
De Souza et al.^40^	Ecological study	TB mortality rate, obtained by national databases^41^	Brazil	–	Yes	UMIC
Reis-Santos et al.^42^	Longitudinal database study	TB cure; broader clinical and social determinants of TB treatment outcomes	Brazil	Mixed	Yes	UMIC
Contreras et al.^43^	Cohort	Socioeconomic needs of recipients of the social protection program “TB Cero”; how “TB Cero” social protection intervention addresses socioeconomic needs through qualitative evaluation	Peru	Peri-urban	No	UMIC
Ngamvithayapong-Yanai et al.^44^	Observational	Treatment outcomes	Thailand	Urban	Yes	LMIC
Diaw et al.^45^	Observational	Treatment outcomes; retention of recipients enrolled in program	Senegal	Rural	Yes	LIC
Wingfield et al.^46^	Cohort study and RCT	Quantify prevalence of catastrophic costs; national TB control program-confirmed TB cure in people with TB	Peru	Urban	Yes	UMIC
Lutge et al.^47^	Unblinded cluster RCT	Treatment outcomes; loss to follow-up and treatment failure rate	South Africa	Mixed	Yes	UMIC
Carter et al.^48^	Quasi-experimental	TB treatment success	Brazil	Mixed	Yes	UMIC
Wei et al.^49^	Quasi-experimental	Cost to person with TB‡; Cost-effectiveness of the social protection program	China	Urban	No	MIC
Wingfield et al.^50^	RCT	Catastrophic costs	Peru	Urban	Yes	UMIC
Wingfield et al.^51^	RCT	Initiation of TB preventive therapy; treatment success	Peru	Urban	Yes	UMIC
Ukwaja et al.^52^	Qualitative	Recipients’ experience of social protection intervention	Nigeria	Urban	Yes	UMIC
Orlandi et al.^53^	Qualitative	Perceived influence of social incentive on treatment adherence among healthcare professionals	Brazil	Urban	Yes	UMIC
George et al.^54^	Qualitative	Analysis of support services available to people with TB	India	Rural	Yes	LMIC
Patel et al.^55^	Mixed methods	Receipt of cash transfer; time to receipt of first cash transfer	India	Urban	Yes	LMIC
Yin et al.^56^	Mixed methods	Treatment outcomes; TB treatment adherence§	China	Urban	Yes	UMIC
Li et al.^57^	Mixed methods	Access to TB diagnosis and treatment; affordability of TB treatment to person with TB	China	Urban	Yes	UMIC
Xiang et al.^58^	Mixed methods	Reimbursement of out-of-pocket costs; catastrophic health expenditure¶	China	Rural	Yes	UMIC
Sripad et al.^59^	Mixed methods	Recipients’ perceptions of social protection program activities available to them; TB treatment adherenceǁ	Ecuador	Mixed	No	MIC

HBC = high-burden country; LMIC = low- and middle-income countries; MIC = middle-income countries; RCT = randomized controlled study; TB = tuberculosis; UMIC = upper middle-income countries.

* Treatment default was defined as anyone who missed treatment for more than 60 days per WHO standards.

† Financial hardship = total costs exceeding 20% of preillness annual household income and/or relying on a negative financial coping strategy (i.e., taking a loan or selling assets); and/or total costs that are impoverishing (incurring total monthly costs that pushed preillness monthly household income per capita below Brazil’s 2016 poverty line [USD 48.6 per month]).

‡ Patient costs = defined as direct medical (clinics, medicines, tests) and nonmedical (travel, food) out-of-pocket payments.

§ Adherence = taking medications 26 days per month up for up to 24 months.

¶ Catastrophic health expenditure was defined as 10% of annual family income.

ǁ Adherence was measured using interruption; anytime during the entire treatment period that two doses of treatment were missed for at least 2 weeks but less than 2 consecutive months.

**Table 3:** Characteristics of gray literature

Author (year)	Document type	Document title	Country	TB HBC	Income classification
Loveleen et al.^60^	Report	An Assessment of the Social Protection Needs and Gaps for Workers in Informal Employment in Myanmar	Myanmar	No	LMIC
Mahadevia^61^	Working paper	Decent Work in Ahmedabad: An Integrated Approach	India	Yes	LMIC
World Bank^62^	Report	The State of Social Safety Nets 2015	Tajikistan	Yes	LMIC
Spray^63^	Report	Leveraging Social Protection Programs for Improved Nutrition: Compendium of Case Studies Prepared for the Global Forum on Nutrition Sensitive Social-Protection Programs	Democratic Republic of Congo	Yes	LMIC
WHO^64^	Report	National Strategic Plan for Ending TB 2020–2024, Timor-Leste	Timor-Leste	Yes	LMIC
Nurova^65^	Project report	Support for Tuberculosis Patients and Their Families Standard Project Report 2016	Tajikistan	Yes	LMIC
WFP^66^	Report	Regional Bureau for Southern Africa	Lesotho	Yes	LMIC
			Madagascar	No	LIC
			Eswatini*	No	LMIC
			Zambia	No	LMIC
WFP^74^	Project report	Supporting Transition by Reducing Food Insecurity and Undernutrition Among the Most Vulnerable	Myanmar	No	LMIC
WFP^67^	Project report	Standard Project Report 2015; World Food Programme in Congo	Democratic Republic of Congo	Yes	LMIC
WFP^41^	Project evaluation	Responding to Humanitarian Needs and Strengthening Resilience to Food Insecurity	Zimbabwe	Yes	LMIC
Foster^68^	PhD thesis	Structure and Agency in the Economics of Public Policy for TB Control	South Africa	Yes	UMIC

HBC = high-burden country; LMIC = low- and middle-income countries; MIC = middle-income countries; TB = tuberculosis; UMIC = upper middle-income countries; WFP = World Food Programme.

In contrast with peer-reviewed publications included in this scoping review, gray literature lacks clear definitions of categories described in Table 2, reflecting data collected in peer-reviewed publications (author, study design, study outcomes).

* Referred to in study as its previous name, Swaziland.

Articles were exported and managed in Covidence (covidence.org, Australia) and duplicate articles removed. Articles with relevant titles qualified for abstract screening. Abstracts screened as relevant to the topic and meeting eligibility criteria were selected for full text inclusion and review. Reference checking of articles eligible for full text review was conducted to identify additional studies missed in the initial search strategy. Two authors (M. H. and H. T.) were responsible for full text screening to minimize selection bias and enhance reliability and validity of this review. Each investigator screened every article once and, where there was disagreement, a third reviewer (T. W. and P. B. S.) acted as a tiebreaker. All observational, qualitative, interventional, and randomized studies meeting the eligibility criteria and written in English were included. All types of reviews and meta-analyses were excluded due to the inherent bias associated with secondary literature where authors have examined a topic and drawn their own conclusions.

For gray literature, searches were carried out in key repositories identified a priori by the ScR team (the WHO [who.int], International Labour Organization [ilo.org], Word Bank [worldbank.org], World Food Programme [WFP], wfp.org). The gray literature search was conducted on June 14, 2021, using Google Advanced and The Bielefield Academic Search Engine (BASE, Bielefeld University Library, Bielefeld, Germany). Results were exported as .CSV files to Microsoft Excel and stored using the GoogleChrome (Alphabet Inc., Mountain View, CA) extension SEOQuake (Semrush Inc., Trevose, PA). The time frame eligibility was defined based on the introduction of the Millennium Development Goals in 2000. Matching the time frame for gray literature to published literature limited the yield of documents to a very low number and, to accommodate this, the search criteria were expanded. Results were limited to documents written in English, pdf files and documents created between 2000 and 2021. Gray literature identified was deemed relevant and eligible if written in English between 2000 and 2021 and a PDF file that named a social protection program in a TB high-burden country or low- and middle-income country and included details of the intervention recipients. Documents were excluded if there were no details of a social protection intervention for people affected by TB or people living in TB-affected households. Finally, selected articles and documents were retrieved for data extraction. Information was inputted into a data extraction table, stored in Microsoft Excel. The data were synthesized in a combination of narrative and tabular format using simple descriptive quantitative analysis. Data were summarized into categories to facilitate the interpretation of findings and draw meaningful conclusions.^20^

A formal consultation exercise was not carried out within this ScR. Instead, the findings of this ScR were presented and discussed at relevant international partnerships and collaborations including the Social Protection Action Research and Knowledge Sharing network (SPARKS, www.sparksnetwork.ki.se) and related, active WHO Advisory Groups and Task Forces.

Ethics approval was not required for this ScR because primary data were not collected and the secondary data used was publicly available.

## RESULTS AND FINDINGS

**Figure 1.**
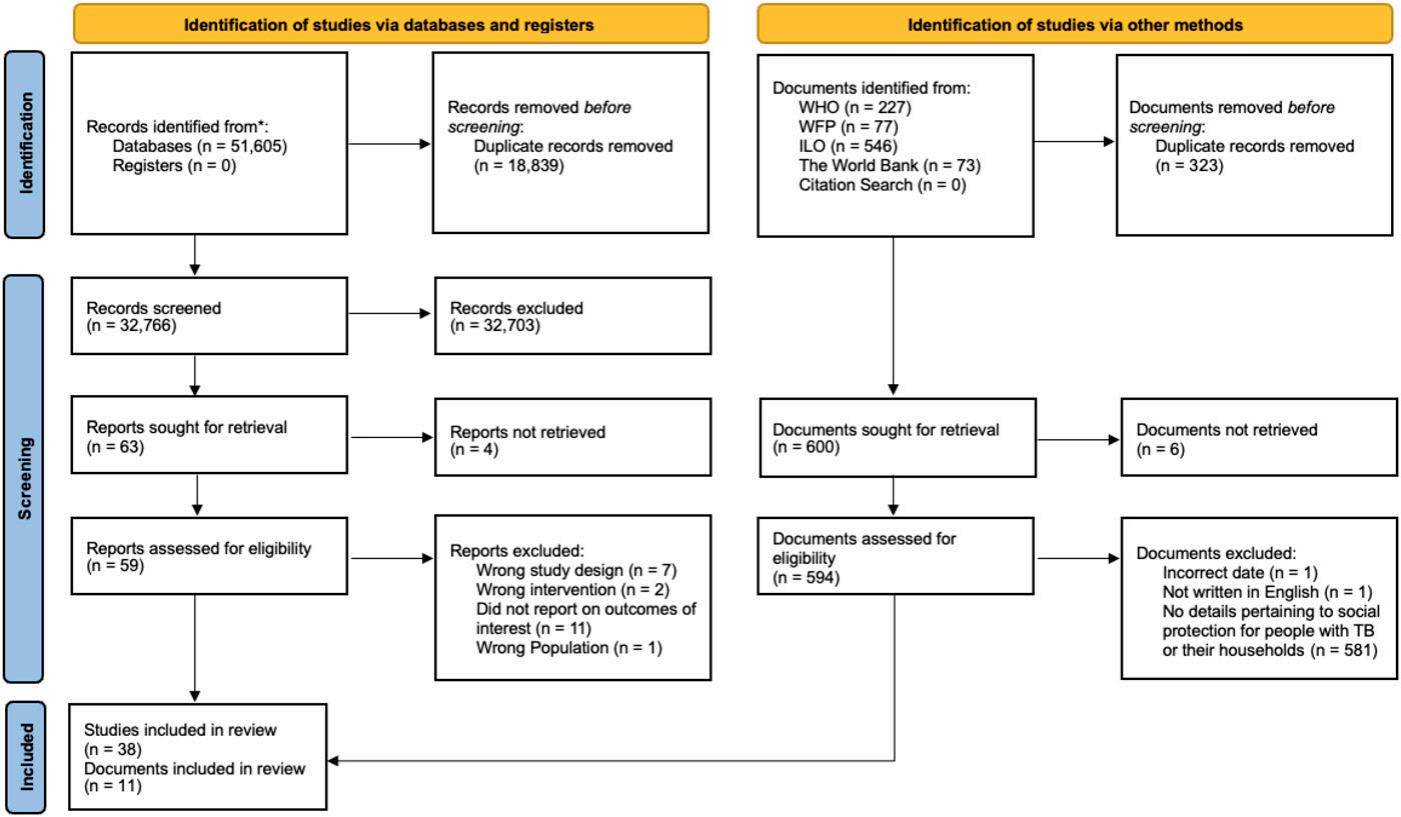
Flow chart of identification, screening, and inclusion of studies included in the scoping review.

The search strategy identified 32,766 discrete articles that were screened according to the eligibility criteria of which 38 were included in this scoping review (Table 2). The gray literature search identified 594 documents that were assessed for eligibility, of which 11 were included in the review (Table 3). A flowchart of the screening and selection process is shown in Figure 1.

### Study characteristics.

Most published studies (24/38, 61%) were observational. There was wide geographic distribution with studies from 15 low- and middle-income countries. Middle-income countries accounted for most included studies with only two being from low-income countries. Thirty-five (92%) studies were from TB high burden countries (HBC). Nearly half (17/38, 45%) of the studies were from South America, and 10 of these were based in Brazil. Most study settings were urban or periurban (21/38, 55%) with four from rural settings and 11 from more than mixed urban and rural settings.

Of the 11 gray literature documents included, 10 were reports from multilateral organizations (WHO, WFP, and ILO) and one was a PhD thesis. These documents described social protection programs from 11 countries, seven of which were TB HBCs.

### Description of social protection programs.

Forty-three social protection programs were identified across the selected studies and are summarized in Table 4. Of the programs identified, 24 were described as TB-specific interventions, 15 were TB inclusive, and four were not classified. These social protection programs were grouped into three distinct categories including financial intervention, food support, and community participation. Financial interventions such as cash transfer tended to be designed to prevent out-of-pocket costs and lost income associated with TB illness and care. Food support and community participation were designed to alleviate the broader impacts of poverty such as food insecurity and malnutrition.

**Table 4:** Summary of characteristics of social protection programs

	Social protection program (*N *= 43)	
	*n*	%
Target recipient group		
All people with TB	17	40
People with MDR-TB	7	16
TB and other diseases	7	16
TB-affected household	5	12
Not defined	3	7
People with TB/HIV	1	2
Migrants with TB	1	2
People with TB and food insecurity	1	2
People with DS-TB	1	2
Social protection type		
Nutritional/food support	13	30
Conditional cash transfer	12	28
A combination of financial and nutritional/food support	9	21
Unconditional cash transfer	5	12
Other financial intervention	2	4.5
Community participation	2	4.5
Funding		
Multilateral organization	16	37
Government	13	30
Mixed funding sources	5	12
Nongovernmental organization	3	7
Other	6	14
TB specific versus TB inclusive		
Specific	26	60
Inclusive	17	40

DS = drug susceptible; MDR = multidrug resistant; TB = tuberculosis.

The eligibility criteria that recipients had to meet to qualify for social protection were similar regardless of the type of social protection offered. Three overarching eligibility criteria were common to all programs: poverty and/or malnutrition, and/or an assessment by a healthcare or social work professional affiliated with the social protection program. Commonly, people with TB had to meet a specified poverty level to be eligible for the intervention. Two programs used other means of assessment including exposure to TB risk factors, and social assessment by a nonstatutory body of socially responsible citizens and volunteers chaired by local government.^26^^,^^69^ For seven of the programs, the eligibility criteria were undefined.

### Financial support interventions.

The type, value, duration and mechanism of financial support and interventions varied greatly. Of the 19 programs that described a financial intervention, 12 used conditional cash transfer, five used unconditional cash transfer and two used a financial intervention other than cash transfer. For example, in Thailand, women of study-defined high socioeconomic status were engaged in social protection programs as financial supporters of those with TB and their households.^44^ Most financial support was given monthly, except for Nikshay Poshan Yojana, a conditional cash transfer program based in India, which made payments once every 2 months, these began only after 2 months of TB treatment is completed.^25^ Although most programs did not stipulate on what the cash or financial support had to be spent, a randomized study in South Africa offered US$15 in the form of a voucher, which could be redeemed in shops chosen by the clinic to monitor spending and prevent purchase of “harmful goods.”^47^ Other programs stated that the cash was intended to be used for nutrition or transport, but spending was in the autonomous control of the recipient.^25^^,^^56^

The size of cash transfers varied greatly from 8 USD in India for an unconditional cash transfer scheme to up to 20,000 USD^55^ in the form of insurance reimbursement in China^57^ (see supplemental material). Minimal evidence exists regarding the proportion of annual income that cash transfers represent and rationale behind the size of transfers, but there was some consensus among reports that the value should be large enough to mitigate against poverty-related TB risk factors and incentivize households to engage with the intervention, while being too small to potentially act as a perverse incentive.

### Nonfinancial support interventions.

Sixteen distinct nonfinancial interventions were identified, the majority (13/16) of which were in the form of nutritional/food support. All nonfinancial support programs were based in Africa and Asia except for the Rocinha Intervention, which provided a supportive community health worker to people with TB in an urban settlement in Brazil.^37^

All nutritional/food support programs were funded by the WFP and targeted a range of TB populations. Six programs offered nutritional/food support to all people with TB, two specifically targeted people with TB and HIV, and two targeted people with DR-TB only. The content of nutrition/food support interventions varied from country to country but consisted of a core basic food parcel containing pulses, cereal, and vegetable oil. Programs aimed to provide the nutritional/food support at daily or monthly frequency and covered a proportion rather than the total food consumption of a TB-affected household.^60^^,^^65^

Community participation programs can be described as social protection or welfare programs that create a supportive network and environment that enables people with TB to adhere to and complete their treatment, adhere to preventive therapy, and avoid deepening impoverishment.^37^^,^^38^^,^^69^ These interventions consisted of a variety of activities including the implementation of educational activities in group settings^37^ and signposting people with TB to appropriate education and free, quality welfare programs.^69^

Some programs incorporated nonfinancial and financial social protection components as either combined or separate interventions. Various examples were identified with a range of approaches including but not limited to electronic vouchers, nutritional supplement, payment of school fees and home utilities, career counseling, transport subsidy, and provision of other materials (e.g., clothing and fuel).^21^^,^^26^^,^^31^^,^^39^^,^^51^^,^^57^^,^^60^^,^^67^

There was a lack of data on direct and indirect costs of delivering financial and especially nonfinancial interventions, which precluded comparative analysis of their budgetary feasibility or impact.

### Funding and resources for social protection.

Funding sources for social programs were variable, but ensuring adequate, suitable funding was reported as essential for success, longevity, and sustainability of social protection programs. The most common source of funding was from multilateral organizations (16/43, 37%). Other sources included NGOs, central government, or a combination of sources. Those funded by central government generally did not report funding issues and had improved staff retention.^37^ Some pilot programs showed smooth transition to more stable, long-term funding despite initially precarious funding. For example, in Thailand, Ngamvithayapong-Yani et al. reported concerns due to receiving initial funding from the Stop TB partnership to provide financial and transportation support to people with TB. However, no long-term funding source was allocated, and when the short-term grant funding ended, wealthy local women were recruited to continue to support the intervention.^44^

### Successes and challenges of implementation.

A review of the articles that provided evaluative commentary on the implementation of social protection interventions identified several barriers to implementation relating to three broad categories: the beneficiary, the provider, and the system by which the program was rolled out.

Challenges included those related to user access and provider shortcomings, authors commonly reported lack of awareness among recipients as a common reason for low coverage of social protection programs.^25^^,^^29^^,^^38^^,^^61^^,^^67^ Others acknowledged there were various administrative and logistical issues reported for the providers with a recurrent issue being delays in instalments of financial support^25^^,^^41^^,^^55^^,^^59^ and hidden or opaque “maintenance” or other charges of banks, which could adversely affect beneficiaries.^47^^,^^51^^,^^52^^,^^55^ Lutge et al. also noted the issues associated with mandatory bank accounts being an eligibility criterion because many individuals in low-resource settings do not have access to a bank and/or do not have their own bank accounts. Hence, the article suggested that the program itself needs to ensure that all potential recipients have a bank account to ensure equity. Lutge et al. also described “street-level bureaucrats” (i.e., individuals who are a subset of a public agency where civil servants have direct contact with the public) who determined who was “worthy” of receiving this intervention; however, the administration period was so long, it often exceeded the length of treatment and consequently this intervention was not widely available.^47^

Some successes were described by authors—for example, the associated emancipation of women through the financial responsibility of managing cash transfers and opening personal bank accounts to receive them.^8^ As well as the overarching acceptability of cash transfer interventions among studied populations, others cited the knock-on beneficial impacts that social protection had on social standing and financial autonomy.^25^^,^^33^^,^^50^

## DISCUSSION

### What social protection is available?

This ScR identified an array of social protection interventions, the language used to describe them, and their intended positive impact on people with TB and their affected households. Overall, 49 documents were identified, of which 43 detailed distinct social protection programs as defined by the World Bank. Most programs were based in middle-income countries with high TB burden. Of these, 24 were TB-specific programs, which focused exclusively on providing financial interventions.

This ScR used a single, recognized social protection definition from the World Bank to inform its search strategy. Despite this, the results showed that a unifying definition of social protection was lacking across studies. Authors used a variety of distinct definitions, and terminology was variable and overlapping to describe the same social protection program activities; this represented a challenge to conducting an effective literature search. For example, seven studies explicitly defined programs as “incentives” where other authors have defined the same interventions as social protection^21^^,^^25^^,^^26^^,^^29^^,^^49^^,^^53^^,^^59^ despite the term not being included in the key words of search strategies of related reviews.^10^^,^^11^^,^^70^ This is a necessary and important distinction because incentives offer reward for treatment rather than reduction of socioeconomic risk that is offered by social protection strategies, as per the World Bank definition used in this ScR.^62^

Consistent with previous studies, this ScR identified that although there was a broad range of TB-specific social protection programs and interventions, most offered financial support, predominantly through conditional cash transfers. Indeed, less than 10% of identified studies reported TB-specific social protection programs that offered nonfinancial support.

Although a variety of subjective values perceived to be relevant to the context and intervention were used to determine eligibility for social protection, poverty was generally assessed using quantitative data on socioeconomic position. The stringent criteria used to target beneficiaries illustrated the complicated relationship between poverty and TB. However, it also highlighted that design and implementation of relevant national policy should be informed by an understanding of the poverty-related socioeconomic barriers that potential recipients face to effectively access social protection programs.^47^

### Successes and challenges of implementation.

Most studies were designed to measure success of interventions quantitatively based on the single outcome of TB treatment outcomes or TB treatment success. A minority of studies reported other outcome measures such as catastrophic costs incurrence or catastrophic health expenditure. However, some identified studies did narratively report implementation successes, which revealed some cross-cutting factors. Secure, adequate, and sustainable funding with robust infrastructure was reported as essential for successful implementation of social protection interventions.^71^ Importantly, these factors dictate both *what* programs people with TB and their households can access and also *how* they are able to access these programs. Further, adequate funding will be necessary to ensure cash transfers sufficiently mitigate the costs associated with TB illness and care. Rohit et al.^25^ and Wingfield et al.^72^ included evaluative commentary relating to charge-free, appropriate bank accounts to support sustainable cash transfer to prevent delay in transfer and reduce risk of theft or fraud.

Although the ethos of cash transfers is to promote equitable access to TB treatment services,^73^ this ScR demonstrated that there are several barriers to achieving equity in resource-limited settings.^47^ This is particularly the case if a program requires bank or electronic transfer of funds or distribution of food baskets, which can be logistically complex and difficult to deliver to underserved groups. Lack of political will, commitment, and sustainable long-term funding—all of which were considered to be out of the control of those delivering non-governmental social protection programs—were identified as threats to the longevity of interventions.

Ultimately, despite social protection being a key feature of the End TB Strategy, the dearth of pragmatic operational and implementation evidence or practical guidance and policy on social protection for people with TB and their households needs to be addressed, particularly with respect to delivery of social protection as part of broader mixed, integrated, or nonfinancial interventions.^7^ Realistic operationalization of social protection programs is discussed by Bustos et al., who noted that successful social protection programs rely on a network of groups in socio-political, relational, and operational contexts.^71^ In light of this, better process evaluation and implementation research is required in this field to support integration of social protection into routine practice and scale-up at national level.

### Recommendations for the design of SR and MA.

Because this work was conducted to inform a systematic review, frequent meetings were held to refine the systematic review search strategy and inclusion criteria iteratively based on the interim findings of the scoping review. The gray literature review ensured that the ScR search strategy was as broad as possible and captured global and regional policy and practice documents that may not have been peer reviewed. The ScR led to some refinement and streamlining of the outcome measures to be used in the systematic review given that several of them, including food insecurity and multidimensional poverty indices, were not measured in any of the included studies. Additionally, the ScR suggested that a narrative synthesis analysis to examine the qualitative outcomes of studies included in the proposed meta-analysis may be of benefit to contextualize interpretation of their quantitative findings.

Descriptive narrative in this ScR pertaining to operational challenges and pragmatic implementation of social protection discovered valuable evaluative comments and revealed opportunity for formal analysis of the characteristics required for successful programs. Numerical assessment of thriving programs in the systematic review could offer a “blueprint” of updated guidance to improve program integration.

### Strengths and limitations.

This ScR was planned to minimize the risk of bias and maintain high quality. The clear, recognized World Bank definition of social protection that was used allowed for a careful selection and high yield of papers, which adds much value in terms of standardizing the language used to describe social protection as per the World Bank definition and how this relates to existing interventions and studies. Although social protection has been part of the global health agenda since the beginning of the millennium, restricting the search from 2012 onward was a necessary limitation. Although some interventions may have been omitted, conclusions have been drawn from a large, broad yield of papers and therefore are likely representative of any additional social protection programs. The findings of this ScR clearly show that the terminology surrounding social protection is ill defined and unclear. Literature could have been missed in the review because the search strategy did not capture all the terminology used to describe social protection programs.

## CONCLUSION

There remains a dearth of high-quality pragmatic trials, effectiveness implementation trials, and rigorous mixed-methods studies in this area, which are essential for assessing feasibility and impact of social protection programs. Such studies provide valuable information to guide policy and decision makers. Nevertheless, this ScR demonstrates the range of designs and mechanisms by which social protection can be distributed to mitigate against the socioeconomic impacts of TB. These findings have informed design and implementation of an ongoing systematic review to evaluate the impact of social protection on TB, health, and socioeconomic outcomes.

## Supplemental files


Supplemental materials
