# Supplementary material for: Social Protection Interventions for TB-Affected Households: A Scoping Review
Source: Am J Trop Med Hyg. 2023 Feb 20;108(4):650–9. doi: 10.4269/ajtmh.22-0470 (PMC10076998; doi:10.4269/ajtmh.22-0470)
Supplement: Supplementary file 1 [file tpmd220470.SD1.pdf]

Details of Social Protection Identified

Programme Name, Country	Type of Social Protection	Social Protection Intervention	Beneficiary	Eligibility criteria	Funding	Conditions	TB Specific
Bolsa Familia Programme (BFP), Brazil	Conditional Cash transfer	Benefits vary from US$18 to US$175 per month.	TB-affected households.	Families defined as poor (per capita monthly income US$25.60-51.20) with pregnant or lactating women or children and adolescents aged 17 years or younger<br>OR<br>All extremely poor families (per capita	Government of Brazil	Attendance at prenatal and postnatal monitoring sessions.<br>Access to nutrition and vaccination monitoring for patients' children aged 0-7 years	No

				monthly income <US$25.60).		school attendance	
CRESPIT, Peru	Mixed Intervention	Ranging from $0-$230 (average $172). Depending on if conditions were met ‘optimally’ or ‘acceptably’.<br><br>High protein food basket, home visits, TB workshops and TB clubs.	TB-affected households.	Newly diagnosed patients with TB from study-site health posts and their household members.	Joint Global Health Trials.	Screening for TB and MDR-TB. Adherence to TB treatment or TB-preventive therapy. Engage with CRESIPT social activities.	Yes
Ecuador's national monetary	Conditional Cash transfer	$240 each month	Patients with DR-TB	People with DR-TB registered with the Ecuador NTP in 2011.	Government of Ecuador	Adherence to DR-TB treatment	Yes

incentive program for drug-resistant TB, Ecuador						(defined as taking medications on 26 days per month for up to 24 months)	
Global Fund programme, China	Conditional Cash transfer	To cover transportation costs (US$10) and nutrition support (US$10)	Adults with confirmed MDR-TB living in poverty.	Adults with confirmed MDR-TB living in poverty.	Global Fund Programme	Not defined	Yes
Kenya National Tuberculosis Leprosy and Lung	Food/Nutrition Support	3 types of support. May qualify for one, a combination, or all three of the following interventions: (1)	Those with TB who are severely	Severe undernourishment as confirmed by nutritional assessment.	Public funding	Not defined	Yes

disease programme, Kenya		Nutrition education and counselling (2) fortified blended food (3) Vitamin A and pyridoxine	undernourished				
Kumar Raajratna Bhimrao Ambedkar Vaidakiya Sahay Yojana (KRBAVSY) aka free medical aid	Unconditional Cash Transfer	500 Rupees paid every month.	Registered TB patients under RNTCP	Belong to schedule caste (SC), schedule tribe, socially and economically backward or economically backward class and having an annual income less than Rs. 36,000 in an urban area	Not stated.	---	No

scheme, India							
NCMS, China	Insurance Reimbursement	Ranging from RMB 100,000 to 150,000 yuan	TB patients receiving outpatient treatment	Not defined.	Government of China & Bill and Melinda Gates Foundation	Not defined.	No
Nikshay Poshan Yojana, India	Conditional Cash transfer	1000 Rupees (US$8) paid every two months.	Patients with TB only.	TB diagnosis regardless of age (inc. paediatric patients), household size or poverty level.	Government of India	Once two months of treatment has been completed.	Yes
Psychosocial Support Groups	Non-financial	Food and hygiene parcels assistance in any kinds of	People with MDR-TB	Eligibility is assessed on an individual and intervention awarded	KNCV Tuberculosis Foundation	Not defined.	Yes

(PSSG), Kazahkstan	Intervention	documentation, assistance in preparation of dossier and submission to get state allowance, interim housing, and admission of children in kindergarten free of charge and in occupation. Psychological counselling of patients and relatives.		according to individual need.	(KNCV) in the framework of the United States Agency for International Development (USAID)		
Ramakrishna Mission Free TB	Mixed Intervention	Nutrition support + what was described by	Patients with MDRTB	Undergoing treatment at Ramakrishna Mission Free TB clinic.	Not stated	Not defined	Yes

clinic Integrated patient support system, India		the authors as "cash handouts".					
Regulatory Decree 170/91 of Law 1046, Argentina	Unconditional Cash Transfer	Paid monthly. Amount paid is variable according to minimum salary, treatment time and presence of co-morbidities.	Not defined.	Social Assessment by health team and permanent residence at least 2 years in the province of Buenos Aires and not being covered by any other social assistance programme.	Not stated.	Not defined.	Yes

Southern Health Improvement Samitee (SHIS), India	Food/nutrition support	Nutrition assistance ($10)<br>Monthly allotments of:<br>13kg Rice<br>3kg lentils<br>over the duration of 60-90 days	TB patients	People with smear positive TB living below the poverty line (<$125/day) in two rural districts of West Bengal	Not for profit organisation.	Not defined.	Yes
STOP-TB Italia Onlus, Senegal	Conditional Cash Transfer	20,000 Franc of the French Community of Africa (FCFA). (US$35.8)	TB patients	TB patients undergoing TB treatment.	Not stated	Treatment adherence	Yes
TB Cero/Zero TB, Peru	Mixed Intervention	Mixed: food vouchers, nutritional supplements, transport to health facilities, chest radiography fees	TB-affected households	Patient and/or head of household interviewed to determine if there are unmet basic needs within households.	NGO & Government of Peru	Not stated	Yes

		for TB evaluation, other medical procedures/medications/healthcare supplies, assistance with accessing social services, rent or utilities payments, furniture, or other expenses (e.g. school fees). Home-based treatment support was also offered.		Using a variety of indicators: poverty score, the number of dependents, household income and expenses, and other sources of support.			
The Rocinha	Community	Supervision of treatment and	TB patients	People with TB living in Rocinha.	Not stated	Not defined	Yes

Intervention, Brazil	Participation	implementation of educational activities to establish a supportive network.					
Transportation subsidy initiative (TSI) to poor patients, China	Conditional Cash Transfer	Intended for transport only. 10CNY per round trip up to a total of 60CNY (for newly diagnosed patients) and 80CNY for retreated during the 6-8 months of treatment.	TB patients	Be defined as a ‘poor’ TB patient.	World Bank Loan, UK department for international development	Not Defined	No
Ukraine Red Cross Society Social	Mixed Intervention	Provide DOTS in homes, incentive food interventions, psychological and	TB patients	Patients deemed 'high risk' by TB physician based on 11 self-reported risk factors:	USAID	Not defined.	Yes

Support Programme, Ukraine		career counselling, vouchers for transportation based on client needs.		homeless, unemployed, a health care worker, a migrant, a refugee or immigrant, an ex-prisoner.			
Social Security Board, Myanmar	Mixed Interventio n	Nutritional support and micronutrient supplements and transportation	TB Patients only	Students, self-employed, farmers and employees of companies with 5+ employees. Dependents of employees not covered	% Of wage paid by employer and worker. Co-financed by external donors and NGOs inc. Global Fund.	Not defined.	No

The Construction Worker's Welfare Board, India	Unconditional Cash Transfer	100,000 rupees paid to workers	People with heart disease, kidney ailment, asthma, tuberculosis or AIDS	Proof of having worked at least 90 days in the form of:<br>1) the talati (president of a village)<br>2) contractor<br>3) rural labour commissioner<br>4) labour commissioner<br>5) the directorate of industrial safety and health.<br><br>Also need certificate, residential proof, three	Tax	Not defined.	No

				photos, proof of age and domicile certificate of Gujarat.			
Nutrition-Sensitive Urban Safety Net Program, Republic of Congo	Mixed Interventio n	Allocation of electronic vouchers for CFAF 30,000 ($60). HHs exchange vouchers for 18 months.<br><br>Nutritional supplement - oil fortified with vitamin A and D for the treatment of moderate malnutrition.	TB-affected Household s.	Households with less than $60 income per month and with one of:<br>(a) a PLW:<br>(b) a PLHIV or person with TB under treatment and malnourished or<br>(c) at least 2 school age children not attending school	World food programme (40%)<br>Government of the Republic of Congo (60%)	- adhere to ARV or DOT<br>- Attent prenatal and postnatal monitoring for PLW and vaccinate children<br>- reintegrate children in school and ensure attendance.	No

TB pension, Timor-Leste	Conditional Cash Transfer	US$150 per month to TB patients	All TB patients (inc. DSTB)	Not defined.	Government of Timor-Leste	Not defined.	Yes
Food Basket, Timor-Leste	Nutrition Support	Rice, pulses, oil, milk, and eggs provided on a monthly basis	All TB patients (inc. DSTB)	Not defined.	Government of Timor-Leste	Not defined.	Yes
Travel Support, Timor-Leste	Conditional Cash Transfer	US$60 for travel support for 6 visits provided in 3 installments at treatment initiation, end of intensive treatment phase and end of treatment	All TB patients (inc. DSTB)	Not defined.	Government of Timor-Leste	Not defined.	Yes

Support for Tuberculosis Patients and Their Families, Tajikistan	Food/Nutrition Support	72kg of fortified wheat flour, 2.7kg of fortified vegetable oil, 7.2kg of pulses and 900g of iodised salt on a bimonthly basis to cover 80% of the required caloric needs of TB patients and 2 family members. (families expected to cover the other 20%)	TB-affected households.	TB patients registered in the DOTS programme. (inc. children)	World Food Programme	Not defined.	Yes
Nutrition support to malnourished children	Food/Nutrition support	Nutrition support to malnourished children and other vulnerable groups in Lesotho	TB patients.	Undergoing TB treatment.	World Food Programme	Not defined	No

and other vulnerable groups in Lesotho, Lesotho							
Programme National De La Nutrition Communautaire, Madagascar	Food /Nutrition support	Monthly ration of super cereal (children under 5 receive oil and sugar additionally)	TB outpatients	Not defined	World Food Programme	Not defined	No
Integrated Management and Acute Malnutrition (IMAM)	Food/Nutrition Support	Supplementary feeding of super cereal, oil puleses and cereals given to outpatients at health centres	TB and HIV patients	Adults who are moderately acute malnourished on ART or TB clients and their households.	World Food Programme	Not defined	No

programme, Eswatini				Moderately acutely malnourished Children 6-59 months and PMTCT/antenatal care clients and their households.			
Food by prescription and counselling for PLHIV and TB patients, Myanmar	Food/Nutrition Support	Individual daily client ration of super cereal; monthly household ration of maize meal, pulses, and vegetable oil. (Corresponds to half of the daily energy requirements for a	MDR-TB patients	Not detailed	World Food Programme	Not defined	No

		household size of six for a month)					
Democratic Republic of the Congo *	Food/Nutrition Support	Daily rations of 250g super cereal and 25g of vegetable oil.	MDRTB patients	Not detailed	World Food Programme	Not defined.	No
ZUNDAF Nutrition Flagship Programme, Zimbabwe *	Food/Nutrition Support	Food intervention and/or voucher distributed	People with TB who are food insecure	Not detailed	World Food Programme	Not defined.	No
Zambia	Food/Nutrition Support	Mobile delivery tracking system using electronic vouchers. Voucher valued at US$25 per household	HIV and TB patients.	Those on ART or TB treatment and children under 24 months.	World Food programme	Attendance at health clinics	No

		per month for 8 months.<br><br>Food basket of cereal meal, pulses and vegetable oil. Selected food insecure and vulnerable households receive micro-nutrient blended food.					
Zimbabwe	Cash Transfer	Either cash or voucher (value not defined)	TB and HIV patients.	Malnourished clients under ART, Pre ART, TB and PMTCT treatment	World Food Programme	Not defined.	No

South Africa	Unconditional Cash Transfer	A voucher (US$15) offered monthly on collection of their treatment for a maximum of 8 months.	Individuals who are unable to work due to a disabling illness	Assessment by a doctor to determine disabling illness preventing work.	South African Socoial Security Agency	Adherence to treatment	No
South Africa	Conditional Cash Transfer	A voucher (US$15) offered monthly for a maximum of 8 months.	Adult and paediatric TB patients	Must have a diagnosis of pulmonary DS-TB and attending intervention clinics.	Research Programme of the National Department of Health (South Africa), the Tuberculosis	Issued on collection of their treatment	Yes

					control assistance program (TB CAP), (the Netherlands, through the South African National TB Directorate) and the Wellcome Trust (United Kingdom)		
Liberia	Mixed Interventio n	Socioeconomic assistance program with three core	HIV, TB and Leprosy	Diagnosis of HIV, TB or Leprosy. Assessment by the	NGO	Not stated	No

		components: (1) transportation reimbursement; (2) food support interventions (sardines, beans rice, vegetable oil; and (3) additional social assistance. All HIV, TB and leprosy patients are eligible for components one and three.<br><br>Additional support in the form of cash	patients eligible for components 1 & 3. Component 2 is TB specific.	Social Protection Officer for additional support.			

		transfer for safe housing, school fees or auxiliary nutritional interventions if patients lack familial support.					
Nigeria	Conditional Cash Transfer	Cash transfer monthly US$15 for 6 months (up to a total of US$90)	DS-TB Patients	Patients participating in first line anti-tuberculosis treatment (DR-TB not eligible) in Ebonyi State, Nigeria.	National TB and Leprosy Control Programme. Ebonyi State Nigeria.	Not stated.	Yes
China	Mixed Interventio n	Capped treatment cost, insurance reimbursement. Capped total treatment intervention cost at	MDR-TB Patients	Patients with multidrug-resistant or rifampicin- resistant tuberculosis who were consecutively	Bill and Melinda Gates Foundation	Unconditional.	Yes

		US$4644. Insurance reimbursement and project subsidies limited patients' expenses to 10% of charges for services within the intervention. Patients given transportation subsidy of $15·50 for each outpatient visit to hospital Health-care providers at community health centres, township health centres, and		diagnosed in a 12-month period after the programme began.			

		village clinics given a total of $15·50 per month per patient for providing DOT.					
China	Cash Transfer	Financial subsidy (RMB 1080/US$170) and transport incentive (RMB 80/US$30).	TB Migrant Patients	Poverty assessment: households with an income < RMB 840 OR stopped working due to infection of TB OR housing rental is oer 1/3 of household income.)	Communicable disease research consortium	Not defined	Yes
Center for Sharing, Thailand	Financial Support	Women's organizations whose members were of high socioeconomic status	TB patients	Eligible patients were identified using a screening questionnaire: (1) Do	Stop TB partnership for initial funding.	Not defined.	Yes

		supported impoverished people with TB by providing social and economic support. People with TB received financial support and home visits (social support) In each visit, four to six volunteers visited the patient at home. The volunteers usually brought milk, eggs, clothes, and cash to support the patient.		you have health insurance? (2) Do you have less than US$ 3 for the whole family or did you experience a food shortage during the last month? (3) Are you able to pay for travel to the TB clinic? (4) Do you have family members, friends or relatives to help when encountering a financial crisis?" Criteria for home support included:			

				extremely poor TB patients, living alone, living with elderly caregivers, or being isolated from the community and patient consent to home visit."			
Moldova	Mixed Intervention	(1) Small cash [20EUR] Paid monthly during out-patient treatment (stopped if treatment interrupted for >5 days)<br>(2) Bigger cash [92EUR] Paid once at the end of treatment	(1) All smear positive patients (including those with MDRTB) after	All TB patients in Moldova (excluding Transnistria)	(1-3) Global Fund<br>(4-9) National Health Insurance Company (NHIC)	Not detailed.	Yes

		(3) reimbursement of transport cost [cost of tickets] paid either daily OR monthly during outpatient treament<br>(4) reimbursement for transport cost [cost of tickets] paid daily during out patient treatment (only intensive phase)<br>(5) Reimbursement of transport cost [cost of tickets] paid weekly during outpatient	hospital discharge<br>(2) All patients who score >5 on social assessme nt<br>(3) All smear positive patients (including those with MDR TB)		(10) Local administratio ns		

		treatment (only intensive phase) (6) Reimbursement for transport cost [cost of tickets or 4.8EURO lump sum] paid monthly during out-patient treatment (only intensive phase) (7) Vouchers for food hygiene and products]2.2 EUR] paid dairly during out-patient treatment (only intensive phase)	after hospital discharge (4) New smear negative patients living in urban areas (5) New smear negative patients living in rural areas				

		(8) Vouchers for food hygiene and products [15.4 EUR] paid weekly during out-patient treatment (only intensive phase, stopped if treatment interrupted for >3 days)<br>(9) Vouchers for food hygiene and products [61.6 EUR] paid monthly during out-patient treatment (only intensive phase, stopped if treatment	(6) New smear negative patients living in rural areas<br>(7) New smear negative patients living in urban areas<br>(8) New smear negative				

		interrupted for >5 days)<br>(10) Other support [clothes, wood etc] paid once	patients living in rural areas<br>(9) New smear negative patients living in rural areas<br>(10) All Patients				
Treatment Support Group (TSG), India	Communit y Participatio n	Socially responsible citizens and volunteers provide social support to each needy TB patient: access to	All TB patients	Need assessment by medical officer, multipurpose health worker, and DOT provider.	Not detailed.	None.	Yes

		information, free and quality services and social welfare programs, empowering the patient for making decision to complete the treatment successfully.					
Nutritional Supplementation for TB patients, India	Food/Nutrition Support	Variable but for example: Kits consisting of 5 kg rice, 5 kg pulses, oil, jaggery were given.	MDR-TB patients only.	Not detailed	Panchayats [Local Self Govern-ment] or Philanthropists or NGOs	Not detailed	Yes.

Trasportation support, India [54]	Conditional Cash Transfer	Additional Rs500/per month as travel allowance for their twice months visits	TB patients.	Not detailed.	Not detailed.	Not detailed.	Not detailed.
* Interventions employed in humanitarian settings							

Glossary of Key Terms

Beneficiary	The individuals or households who are targeted to benefit from the social protection intervention. This can include or go beyond the recipient.
Catastrophic Costs	Total TB care costs, that is direct medical (i.e. consultations, tests, pills) and non-medical (i.e. food, travel, accommodation) out-of-pocket costs and lost income exceeding 20% of a household's annual pre-TB income, exceeding 20% of annual household income
Conditional Cash Transfer	Direct, regular, and predictable transfers that are given with the requirement that the recipient meets certain conditions.
Direct costs	Costs that are directly attributable to patient care and paid for out-of-pocket. Can include direct medical (i.e. consultations, tests, pills) and non-medical (i.e. travel, food, accommodation) out-of-pocket costs)
Financial Intervention	Any social protection intervention in the form of a financial payment to the recipient.
Indirect costs	Costs incurred during treatment or illness that are not because of medical management of the disease. Indirect costs include lost income and productivity.
Multilateral Organisation	Organisations formed by three or more nations to work on issues relevant to them.

Psychosocial Support	Support given to help meet the mental, emotional, social, and spiritual needs of people with TB and their households.
Recipient	Individuals or households who receive the social protection intervention.
Social Protection	Social protection systems help individuals and families, especially the poor and vulnerable, cope with crises and shocks, find jobs, improve productivity, invest in the health and education of their children, and protect the aging population. Social protection programs are at the heart of boosting human capital for the world’s most vulnerable. They empower people to be healthy, pursue their education, and seek opportunity to lift themselves and their families out of poverty.
TB Inclusive	Interventions in which having tuberculosis is an inclusion criterion for the programme but may not be the only one.
TB Sensitive	Interventions that can potentially affect tuberculosis epidemiology and control, but it is not the primary design.
TB Specific	Interventions targeted specifically at households or individuals affected by TB.

Unconditional Cash Transfer	Direct, regular, and predictable transfers that do not have any requirements in terms of how they are spent or conditions to be received.

Full Search Queries

PubMed (includes MEDLINE) search query

("2012"[Date - Publication] : "2021"[Date - Publication]) AND (Tuberculosis[MeSH] OR Tuberculosis[Title/Abstract] OR TB[Title/Abstract] OR "Mycobacterium tuberculosis" [Title/Abstract] OR "Pulmonary TB" [Title/Abstract] OR "TB affected" [Title/Abstract] OR "TB infected" [Title/Abstract] OR "TB patients" [Title/Abstract] OR "Drug-resistant TB" [Title/Abstract] OR "TB individuals" [Title/Abstract] OR "TB affected households" [Title/Abstract] OR "TB prevalent" [Title/Abstract] OR "Pulmonary tuberculosis" [Title/Abstract] OR "Pulmonary TB" [Title/Abstract] OR PTB[Title/Abstract]) AND (Social protection[Title/Abstract] OR "Social safety net" [Title/Abstract] OR "Socioeconomic support" [Title/Abstract] OR "Social support" [Title/Abstract] OR "Economic support" [Title/Abstract] OR "Financial support" [Title/Abstract] OR "Cash transfers" [Title/Abstract] OR "Conditional cash transfers" [Title/Abstract] OR "Unconditional cash transfers" [Title/Abstract] OR "supplementary feeding programmes" [Title/Abstract] OR "food stamps" [Title/Abstract] OR "vouchers" [Title/Abstract] OR "coupons" [Title/Abstract] OR "in-kind transfers" [Title/Abstract] OR "school supplies" [Title/Abstract] OR "uniforms" [Title/Abstract] OR "Food baskets" [Title/Abstract] OR "Food rations" [Title/Abstract] OR "Social risk management" [Title/Abstract] OR Transportation[Title/Abstract] OR "Government financing" [Title/Abstract] OR Reimbursement[Title/Abstract] OR "Support groups" [Title/Abstract] OR Education[Title/Abstract] OR "Community support" [Title/Abstract] OR Welfare [Title/Abstract] OR Support[Title/Abstract] OR Intervention[Title/Abstract] OR Program[Title/Abstract] OR Scheme[Title/Abstract] OR Policy[Title/Abstract] OR Assistance[Title/Abstract] OR Livelihood support[Title/Abstract] OR

Impact[Title/Abstract] OR Affect[Title/Abstract] OR Effect[Title/Abstract] OR Association[Title/Abstract] OR Associated[Title/Abstract] OR Consequence[Title/Abstract] OR incentive[Title/Abstract] OR enabler[Title/Abstract]) AND (Treatment[Title/Abstract] OR Outcome[Title/Abstract] OR Success[Title/Abstract] OR Rates[Title/Abstract] OR Unsuccessful[Title/Abstract] OR Uptake[Title/Abstract] OR Enrollment[Title/Abstract] OR Adherence[Title/Abstract] OR Cured[Title/Abstract] OR Completed[Title/Abstract] OR Treated[Title/Abstract] OR "Follow-up" [Title/Abstract] OR "loss to follow-up" [Title/Abstract] OR Relapse[Title/Abstract] OR Recurrence[Title/Abstract] OR "Adverse outcome" [Title/Abstract] OR "Diagnostic pathways" [Title/Abstract] OR "TB testing" [Title/Abstract] OR "Quality of life" [Title/Abstract] OR Default[Title/Abstract] OR "Care cascade" [Title/Abstract] OR Socioeconomic[Title/Abstract] OR Outcome[Title/Abstract] OR "Financial burden" [Title/Abstract] OR "Economic burden" [Title/Abstract] OR "Economic consequences" [Title/Abstract] OR "Social consequences" [Title/Abstract] OR "Socioeconomic consequences" [Title/Abstract] OR "Social impact" [Title/Abstract] OR "Socioeconomic impact" [Title/Abstract] OR Costs [Title/Abstract] OR Expenditure[Title/Abstract] OR Expenses[Title/Abstract] OR Spending[Title/Abstract] OR Catastrophic[Title/Abstract] OR Expenditure[Title/Abstract] OR "Catastrophic costs" [Title/Abstract] OR Impoverishment[Title/Abstract] OR "Coping strategies" [Title/Abstract] OR Poverty[Title/Abstract] OR "Food security" [Title/Abstract] OR "Food insecurity" [Title/Abstract] OR "Loans" [Title/Abstract] OR "Sold assets" [Title/Abstract] OR Dissaving[Title/Abstract] OR Deprivation[Title/Abstract] OR Defray[Title/Abstract] OR Mitigate[Title/Abstract])

Embase search query

('tuberculosis'/exp OR tuberculosis:ab,ti OR TB:ab,ti OR 'Mycobacterium tuberculosis':ab,ti OR 'Pulmonary TB':ab,ti OR 'TB affected':ab,ti OR 'TB infected':ab,ti OR 'TB patients':ab,ti OR 'Patients with TB':ab,ti OR 'People with TB':ab,ti OR 'Drug-resistant TB':ab,ti OR 'TB individuals':ab,ti OR 'TB affected households':ab,ti OR 'TB prevalent':ab,ti OR 'Pulmonary tuberculosis':ab,ti OR 'Pulmonary TB':ab,ti OR PTB:ab,ti) AND (Social protection:ab,ti OR 'Social safety net':ab,ti OR 'Socioeconomic support':ab,ti OR 'Social support':ab,ti OR 'Economic support':ab,ti OR 'Financial support':ab,ti OR 'Cash transfers':ab,ti OR 'Conditional cash transfers':ab,ti OR 'Unconditional cash transfers':ab,ti OR 'food-based programmes':ab,ti OR 'supplementary feeding programmes':ab,ti OR 'food stamps':ab,ti OR 'vouchers':ab,ti OR coupons:ab,ti OR 'in-kind transfers':ab,ti OR 'school supplies':ab,ti OR uniforms:ab,ti OR 'Price subsidies for food':ab,ti OR 'Price subsidies for electricity':ab,ti OR 'Price subsidies for public transport':ab,ti OR 'Public works programmes':ab,ti OR 'Fee waivers for health care':ab,ti OR 'Exemptions for health care':ab,ti OR 'Fee waivers for schooling':ab,ti OR 'Exemptions for schooling':ab,ti OR 'Fee waivers for utilities':ab,ti OR 'Exemptions for utilities':ab,ti OR 'Food baskets':ab,ti OR 'Food rations':ab,ti OR 'Protections against shocks':ab,ti OR 'Social risk management':ab,ti OR Transportation:ab,ti OR 'Government financing':ab,ti OR Reimbursement:ab,ti OR 'Support groups':ab,ti OR Education:ab,ti OR 'Community support':ab,ti OR Welfare:ab,ti OR Support:ab,ti OR Intervention:ab,ti OR Program:ab,ti OR Scheme:ab,ti OR Policy:ab,ti OR Assistance:ab,ti OR Livelihood support:ab,ti OR Impact:ab,ti OR Affect:ab,ti OR Effect:ab,ti OR Association:ab,ti OR Associated:ab,ti OR Consequence:ab,ti OR incentive:ab,ti OR enabler:ab,ti) AND (Treatment:ab,ti OR Outcome:ab,ti OR

Success:ab,ti OR Rates:ab,ti OR Unsuccessful:ab,ti OR Uptake:ab,ti OR Enrollment:ab,ti OR Adherence:ab,ti OR Cured:ab,ti OR Completed:ab,ti OR Treated:ab,ti OR 'Follow-up':ab,ti OR 'loss to follow-up':ab,ti OR Relapse:ab,ti OR Recurrence:ab,ti OR 'Adverse outcome':ab,ti OR 'Diagnostic pathways':ab,ti OR 'TB testing':ab,ti OR 'Quality of life':ab,ti OR default:ab,ti OR 'care cascade':ab,ti OR Socioeconomic:ab,ti OR Outcome:ab,ti OR 'Financial burden':ab,ti OR 'Economic burden':ab,ti OR 'Economic consequences':ab,ti OR 'Social consequences':ab,ti OR 'Socioeconomic consequences':ab,ti OR 'Social impact':ab,ti OR 'Socioeconomic impact':ab,ti OR Costs:ab,ti OR Expenditure:ab,ti OR Expenses:ab,ti OR Spending:ab,ti OR Catastrophic:ab,ti OR Expenditure:ab,ti OR 'Catastrophic costs':ab,ti OR Impoverishment:ab,ti OR 'Coping strategies':ab,ti OR Poverty:ab,ti OR 'Food security':ab,ti OR 'Food insecurity':ab,ti OR Loans:ab,ti OR 'Sold assets':ab,ti OR Dissaving:ab,ti OR Deprivation:ab,ti OR Defray:ab,ti OR Mitigate:ab,ti AND [2012-2021]/py)

Web of Science search query

Note: (Unable to include date in syntax; date set to "2012-2021")

TI=(Tuberculosis OR TB OR "Mycobacterium tuberculosis" OR "Pulmonary TB" OR "TB affected" OR "TB infected" OR "TB patients" OR "Drug-resistant TB" OR "TB individuals" OR "TB affected households" OR "TB prevalent" OR "Pulmonary tuberculosis" OR "Pulmonary TB" OR PTB) AND TI=(Social protection OR "Social safety net" OR "Socioeconomic support" OR "Social support" OR "Economic support" OR "Financial support" OR "Cash transfers" OR "Conditional cash transfers" OR "Unconditional cash transfers" OR "supplementary feeding programmes" OR "food stamps" OR "vouchers" OR "coupons" OR "in-kind transfers" OR "school supplies" OR "uniforms" OR "Food baskets" OR "Food rations" OR "Social risk management" OR Transportation OR "Government financing" OR Reimbursement OR "Support groups" OR Education OR "Community support" OR Welfare OR Support OR Intervention OR Program OR Scheme OR Policy OR Assistance OR Livelihood support OR Impact OR Affect OR Effect OR Association OR Associated OR Consequence OR incentive OR enabler) AND TS=(Treatment OR Outcome OR Success OR Rates OR Unsuccessful OR Uptake OR Enrollment OR Adherence OR Cured OR Completed OR Treated OR "Follow-up" OR "loss to follow-up" OR Relapse OR Recurrence OR "Adverse outcome" OR "Diagnostic pathways" OR "TB testing" OR "Quality of life" OR Default OR "Care cascade" OR Socioeconomic OR Outcome OR "Financial burden" OR "Economic burden" OR "Economic consequences" OR "Social consequences" OR "Socioeconomic consequences" OR "Social impact" OR "Socioeconomic impact" OR Costs OR Expenditure OR Expenses OR Spending OR Catastrophic OR Expenditure OR "Catastrophic costs" OR Impoverishment OR "Coping strategies"

OR Poverty OR "Food security" OR "Food insecurity" OR Loans OR "Sold assets"

OR Dissaving OR Deprivation OR Defray OR Mitigate)
